# Supplementary material for: 3D-architected gratings for polarization-sensitive, nature-inspired structural color
Source: Nanophotonics. 2025 Mar 10;14(5):547–58. doi: 10.1515/nanoph-2024-0657 (PMC11953725; doi:10.1515/nanoph-2024-0657)
Supplement: Supplementary file 1 — Supplementary Material Details [file j_nanoph-2024-0657_suppl_001.pdf]

Research Article

Moisés H. Ibarra Miranda, Lars W. Osterberg, Dev H. Shah, Kartik Regulagadda and Lisa V. Poulikakos*

Supplementary Material for: 3D-architected gratings for polarization-sensitive, nature-inspired structural color

1 Diffraction efficiency dependence analytical study on grating pitch

When modifying the grating pitch, we insert new values for the periodicity (Λ) in Equation 4. It is noteworthy that, as mentioned in Section 2 of the main text, the analytical model in the Raman-Nath regime is inaccurate for smaller Λ, as the thin grating classification no longer holds and numerical methods are needed to model the diffraction efficiency at higher wavelengths (λ) [40]. Therefore, in Figure S1, the λ range in which the model is still applicable to all Λ is generally below ca. 0.5 µm. It is important to note that, regardless of Λ variations, the grating should transmit a high proportion of blue light.

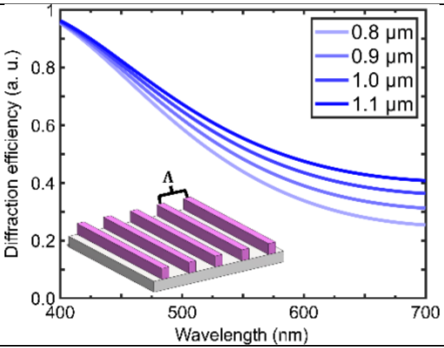

Figure S1. Diffraction efficiency according to the Raman-Nath model (eq. 4-5) for Λ=0.8, 0.9, 1.0, and 1.1 µm plotted for λ, with $w = 200$ nm and $d = 700$ nm. The Raman- Nath model is inaccurate for smaller Λ studied here, as the thin grating classification no longer applies.

*Corresponding author: Lisa V. Poulikakos, Department of Mechanical and Aerospace Engineering, Program of Materials Science and Engineering, University of California San Diego, La Jolla, CA, USA; E-mail: lpoulikakos@ucsd.edu; https://orcid.org/0000-0002-1118-789X
Moisés H. Ibarra Miranda: Program of Materials Science and Engineering, University of California San Diego, La Jolla, CA, USA; E-Mail: moibarramiranda@ucsd.edu; https://orcid.org/0009-0008-7685-974X
Lars W. Osterberg: Department of Mechanical and Aerospace Engineering, University of California San Diego, La Jolla, CA, USA; E-mail: losterberg@ucsd.edu
Dev H. Shah: Department of Mechanical and Aerospace Engineering, University of California San Diego, La Jolla, CA, USA; E-mail: deshah@ucsd.edu
Kartik Regulagadda: Laboratory for Multiphase Thermofluidics and Surface Nanoengineering, Department of Mechanical Engineering, University of California, Berkeley, CA, USA; E-mail: regkartik@berkeley.edu; https://orcid.org/0000-0002-5787-9234

2 Two-photon lithography fabrication

We obtained fused silica substrates (25 × 25 × 0.7 mm^3), polished on both sides, from Nanoscribe GmbH & Co.KG. The substrates are blown with nitrogen before mounting them onto the multi-DiLL sample holder. They are secured in place using sticky tape on the edges. A drop (~30 µL) of IP-Dip photoresist is dispensed at the center of the substrate. Dip printing mode is selected for our work using a 63× objective. A custom GWL code is implemented to print the structures at a desired scan speed (SS) and laser power (LaP). To estimate the optimal values, we performed a parametric sweep with SS ranging from 20000 to 40000 and LaP ranging from 45% to 80% of the total power of 40mW, respectively. Both these parameters define the voxel size, thereby governing the structure height (d) and print quality.

For d measurements, we increased the z-offset so that the grating line could collapse (see Figure 3 in the main text). Further, the z-offset would define the apparent d of each layer in the log-pile structure along with adhesion strength to the substrate. After careful consideration, we decided to set the z-offset to 600 nm to achieve the desired height and ensure structural stability. The length of an individual structure in the log-pile varies from 50 µm to 100 µm depending on the periodicity. Λ values between two individual structures are varied from 600 nm to 1000 nm. Printing is performed in a layer-by-layer approach. Once printing is complete, the samples are removed from the TPL system and immersed successively in propylene glycol methyl ether acetate (PGMEA) followed by isopropyl alcohol (IPA) for 15 min and 2 min, respectively, for development. The samples are then removed from IPA, and dried at ambient conditions until the solvent evaporates completely. The printed substrates are sputter coated with 5 nm Platinum coating whenever visualization under SEM is preferred.

3 Morphological characterization of 3D architected gratings

Scanning electron microscopy (SEM) provided a surface-morphology analysis of the grating structures to evaluate structural conditions. An SEM *Sigma 500* (ZEISS) was utilized to study samples of different periodicity Λ (see Figure S2(a)-(d)), number of layers (see Figure S2(e)-(h)), and pillar height d (see Figure S2(i)-(l)). Imaging conditions included a voltage of 5.0 kV, and magnification of 1470× and 37 730×. The detection was performed by a secondary electron detector (SE2). The sample preparation involved a Ti layer deposition of 5 nm and a 2 cm-long carbon tape that connects the sample surface to the stub to ground it.

Figure S2 shows SEM images of the fabricated structures as fabrication parameters (Λ, number of layers, and d) are varied. In Figure S3(a)-(d), bi-grating structures became smaller as Λ is reduced, covering areas from 40 µm × 40 µm (at Λ=1.1 µm) to 28 µm × 28 µm (at Λ=0.8 µm). In the case of the number of layers study (see Figure S3(e)-(h)), the morphological conditions are less consistent when adding more layers, as observed primarily at the sample edges. Furthermore, the covered areas for 3- and 4-layer bi-gratings are reduced as Λ is unintentionally reduced. This may be attributed to increased mechanical strain within the 3- and 4-layer gratings and the w-d dimensional proportions, which can be explored in future studies. Finally, structures with different heights (see Figure S3(i)-(l)) exhibited consistent morphological conditions as SS and LaP were modified.

For width (w) measurements, higher magnification (37730× magnification) SEM images are taken in a selected area for a bi-grating (d=907 ± 21 nm and Λ=1.1 µm) (see magnification comparison in Figure S3(a,b)). Similarly, this is performed for bi-gratings under Λ variations (ranging from 0.8 µm to 1.1 µm) (see Figure S3(c)-(f)). In Figure S3, w-measurement cuts are shown in pink and blue for the bottom (w_{bottom}) and top (w_{top}) layer, respectively. The data is statistically expressed by average/arithmetic mean ($\overline{w}$) and standard deviation (s_d) using the following equations:

$$\overline{w} = \frac{\sum w_i}{n}, \tag{S1}$$

$$s_w = \sqrt{\frac{\sum (w_i - \overline{w})^2}{n-1}}. \tag{S2}$$

where w_i represents each individual w measurement and n is the total number of measurements. According to Figure S3, w values are relatively comparable, which demonstrate the w-dependence on printing conditions, as these gratings are fabricated with equal SS and LaP.

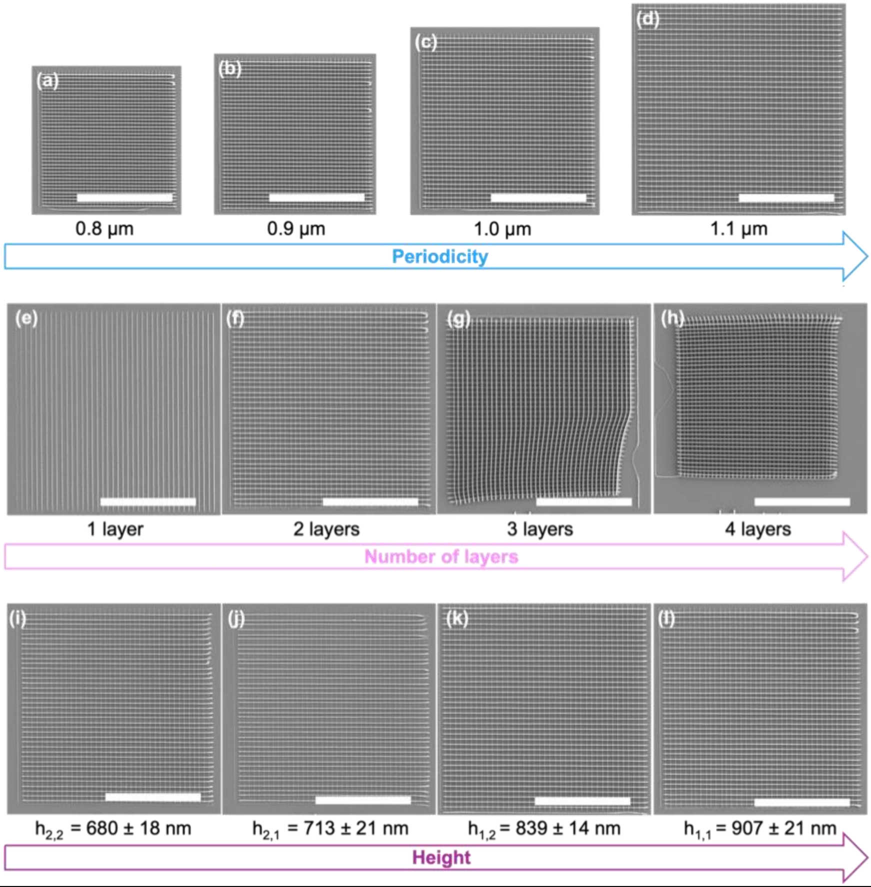

Figure S2. SEM images (1470× magnification) of grating structures varying (a)-(d) Λ (ranging from 0.8 µm to 1.1 µm for a bi-grating), (e)-(h) number of layers (ranging from 1 to 4 grating layers with Λ=1.1 µm) and (i)-(j) d (ranging from 680 ± 18 nm to 907 ± 21 nm with Λ=1.1 µm for a bi-grating). Scale bar: 20 µm.

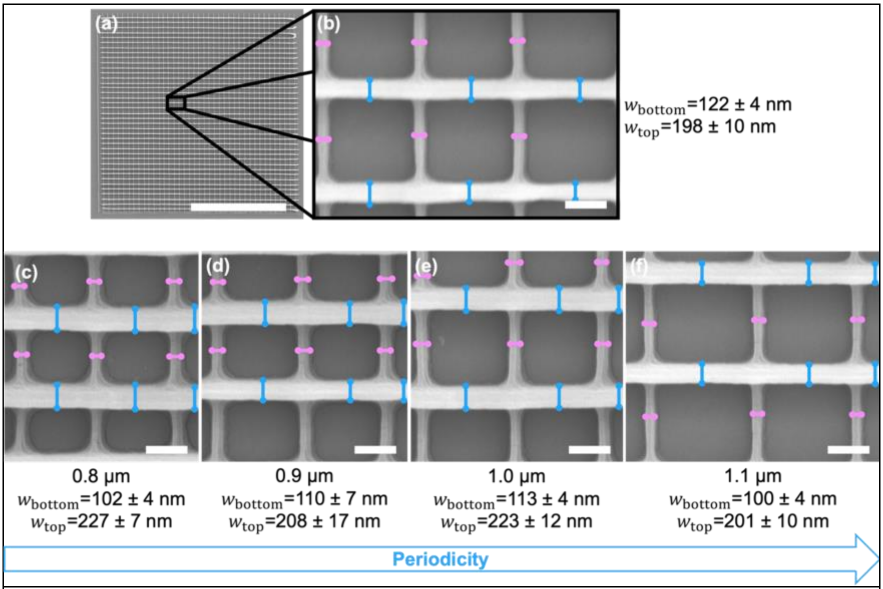

Figure S3. SEM images of a bi-grating (d=907 ± 21 nm and Λ=1.1 µm) at (a) 1470× (scale bar: 20 µm) and (b) 37730× (scale bar: 200 nm) magnifications. (c)-(f) bi-gratings under Λ variations (ranging from 0.8 µm to 1.1 µm) at 37 730× magnification. Pink and blue cuts represent w measurements for w_{bottom} and w_{top}, respectively. Scale bar: 200 nm.

4 Height characterization of 3D architected gratings

Atomic force microscopy (AFM) was implemented to obtain d profiles of bi-grating structures, using a *Park NX20* (Park System) under non-contact mode. Two pairs of samples with the same fabrication conditions were investigated: 1) SS of 30000 µm/s and LaP of 60% and 2) SS of 50000 µm/s and LaP of 65%.

45º-rotated 10 µm × 10 µm areas were scanned at a speed of 0.5 Hz. As shown in Figure S3, two different areas were studied for each sample. Herein, three d profiles from both the top and bottom layer were statistically analyzed, indicating the overall averaged d. Additionally, the specific cuts are represented in the AFM images. Gwyddeon was employed to process 24-bit uncompressed TIFF files of AFM images. Here, we leveled data by mean plane subtraction and shifted the minimum data value to zero. Next, we extracted d profiles along the cuts (see cuts in Figure S4(g,h,o,p,w,x,ae,af)) and find d peaks with a zero-background type. d data was statistically expressed by average/arithmetic mean ($\bar{d}$) and standard deviation (s_d) using the following equations:

$$\bar{d} = \frac{\sum d_i}{n}, \tag{S3}$$

$$s_d = \sqrt{\frac{\sum (d_i - \bar{d})^2}{n-1}}. \tag{S4}$$

where d_i represents each individual d peak.

Ultimately, height measurements were utilized for the height dependence study of structural coloration. The first pair of bi-gratings showed comparable average d values (907 ± 21 nm and 839 ± 14 nm). In contrast, increasing SS and decreasing LaP for the second pair resulted in lower bi-grating d (713 ± 21 nm and 680 ± 18 nm). For both pairs, differences in measurements are attributed to experimental variations during the TPL process, primarily arising when calibrating the sample stage.

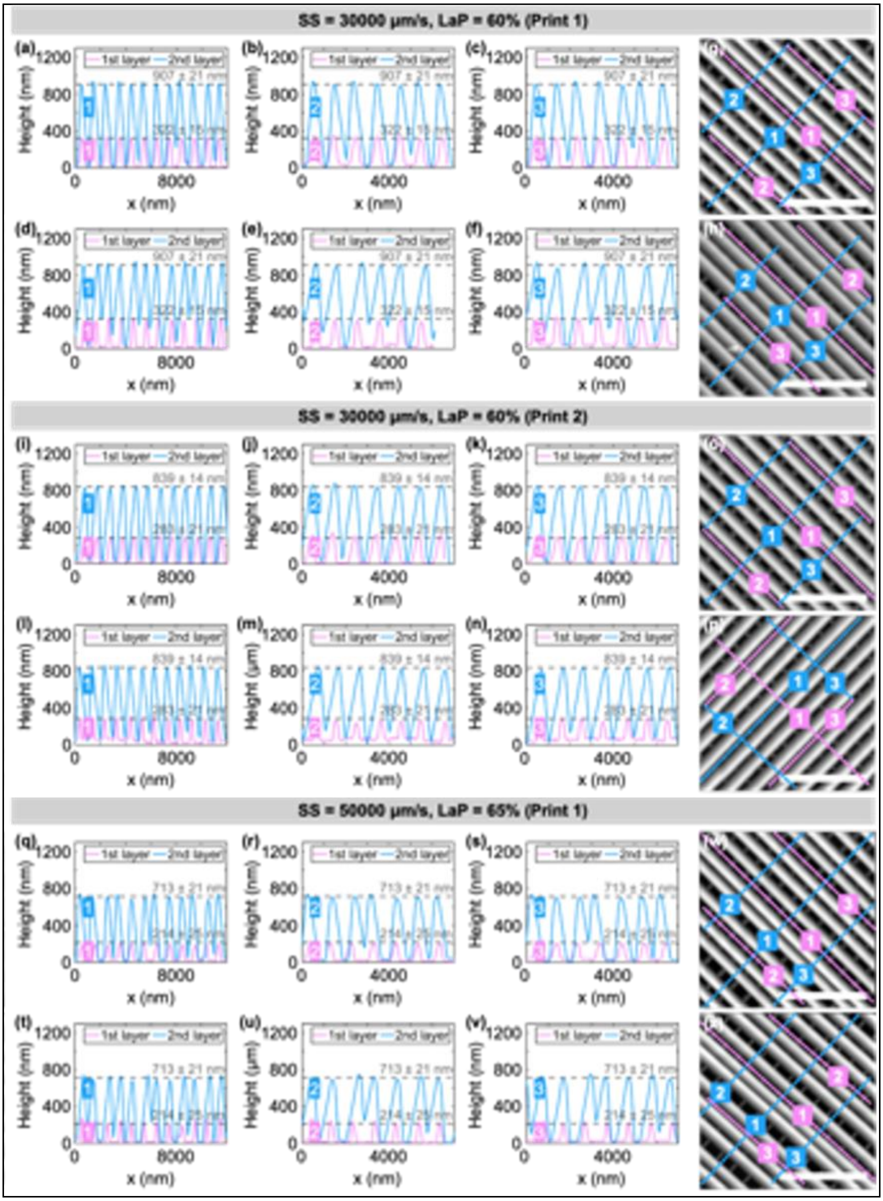

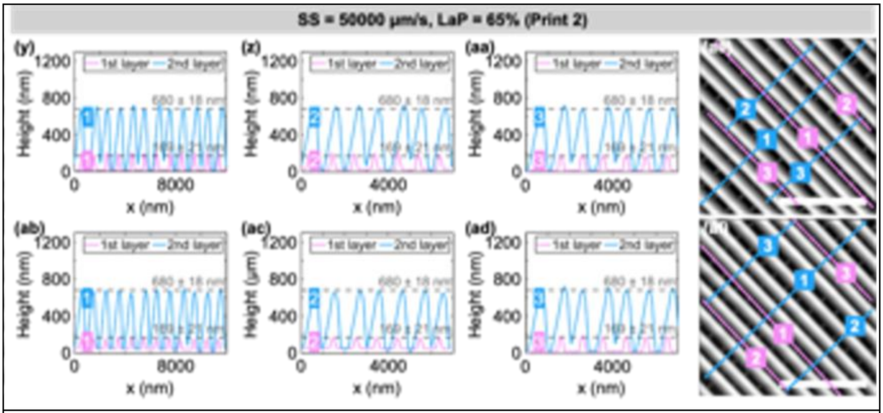

Figure S4. AFM analysis of the layer d. (a)-(f) d profiles and (g,h) 10 µm × 10 µm sectional area with cuts indicated for Print 1 with fabrication conditions of SS = 30000 µm/s and LaP = 60%. (i)-(n) d profiles and ((o), (p)) 10 µm × 10 µm sectional area with cuts indicated for Print 2 with fabrication conditions of SS = 30000 µm/s and LaP = 60% . (q)-(v) d profiles and (w,x) 10 µm × 10 µm sectional area with cuts indicated for Print 1 with fabrication conditions of SS = 50000 µm/s and LaP = 65%. (y)-(ad) d profiles and (ae,ai) 10 µm × 10 µm sectional area with cuts indicated for Print 2 with fabrication conditions of SS = 50000 µm/s and LaP = 65%. Scale bar: 5 µm.

5 Optical characterization

Grating samples were imaged using a Nikon ECLIPSE LV100ND polarized light microscope coupled with a Nikon LV-HL 50W 12V LONGLIFE halogen lamp, a Nikon DS-Ri2 microscope camera, and a 20× objective. For each color channel, relative efficiencies over a 0.0432 μm^2-area in the 8-bit RGB image were calculated as a fraction of the maximum possible intensity value of 255, for the halogen lamp illuminating the sample substrate, i.e. the background signal. This resulted in relative efficiencies of 0.8806, 0.9042, and 0.5091 for red, green, and blue color channels, respectively. Incident polarized light was provided by a D-DP polarizer and an analyzer with a 360° rotary dial. The input polarizer was positioned along the horizontal and vertical for linearly (LP) and circularly polarized light microscopy, respectively. For the azimuthal angle variation study, the stage was rotated clockwise from 0° to 90° in 15°-steps. Incident lamp intensity was consistent throughout the studies. Images were acquired in the NIS Elements Basic Research software and saved as 24-bit uncompressed TIFF files. The pixel size was set to 0.435 µm.

Next, spectra were recorded under the abovementioned conditions with a Princeton Instruments IsoPlane 160 spectrometer and processed in LightField 64-bit Data Acquisition. For the acquisition process, the “Custom Regions of Interest” condition was employed (Bin W = 1, Bin H = 11). 6000 ms was configured as the exposure time, 500 nm as the center wavelength, 500 µm as the grating slit, and the grating was 300 g/mm. Spectra were normalized by dividing the grating transmittance by the transmittance spectra of the lamp at the same incident intensity. The MATLAB smoothdata function was used to smooth the normalized spectra with a Gaussian-weighted moving average filter over a 200 nm window.

The color matching functions of the International Commission on Illumination (CIE) were implemented to calculate the XYZ tristimulus values of the transmitted colors, defined by [1], [2]:

$$X = k \sum_{\lambda} \phi_{\lambda} \bar{x}(\lambda) \Delta\lambda, \quad \text{(S5)}$$

$$Y = k \sum_{\lambda} \phi_{\lambda} \bar{y}(\lambda) \Delta\lambda, \quad \text{(S6)}$$

$$Z = k \sum_{\lambda} \phi_{\lambda} \bar{z}(\lambda) \Delta\lambda, \quad \text{(S7)}$$

where ϕ_{λ} is the spectral distribution for a wavelength λ (ranging between 400-700 nm). Herein, the color-matching functions of a standard colorimetric observer $\bar{x}(\lambda)$, $\bar{y}(\lambda)$, and $\bar{z}(\lambda)$ involve a step-size ($\Delta\lambda$) for wavelength measurements. k, the normalizing factor, is defined so that Y =100 for objects with transmittance equal to 1 over the entire wavelength range:

$$k = \frac{100}{\sum_{\lambda} S(\lambda) \bar{y}(\lambda) \Delta\lambda} \quad \text{(S8)}$$

where $S(\lambda)$ is the spectral distribution of the illuminant. Finally, to find the chromaticity coordinates (x and y), the following equations are used:

$$x = \frac{X}{X+Y+Z} \quad \text{(S9)}$$

$$y = \frac{X}{X+Y+Z}. \quad \text{(S10)}$$

These values are used to represent the specified color on a two-dimensional CIE 1931 color space [3]. In this work, two-dimensional CIE 1931 chromaticity diagrams were generated to visually represent grating transmitted colors in the CIE color space. First, using *Mathematica*'s *ImageMeasurements* and *RGBColor* functions, average colors from optical images were determined in sRGB values (evaluating the three additive colors: red, green, and blue) [4], [5]. Next, the *ChromaticityPlot* function takes the mean sRGB values as input and, delimited by a D50 illuminant white point, plots each average color in the CIE color space. This function automatically converts to coordinates in the CIE 1931 xy chromaticity diagram color space, showing a slice determined by a constant luminance of 0.01 [6], [7].

6 Analysis of polarization dependence in bigratings

In this section we provide additional information on the polarization-sensitive optical response of the studied grating systems by comparing a monolayer grating (d=322 ± 15 nm and Λ=1.0 µm) and a bilayer grating (d=907 ± 21 nm and Λ=1.1 µm) for incident linear polarization (LP) at φ=0° and φ=90°, respectively. Note that the fabrication parameters of the monolayer grating match that of the bottom layer in the studied bilayer grating. At φ=0°, the LP incident light is oriented perpendicular to the monolayer and bottom layer of the bigrating.

Figure S5 shows transmittance spectra of the monolayer (Figure S4(a)) and bilayer (Figure S4(b)) grating for φ=0° and φ=90°, respectively. Insets show SEM images and optical microscope images of the relevant structural color response. In Figure S4(a), the monolayer grating is activated at φ=90°, where the LP orientation is parallel to the gratings, while the response at φ=0° gives rise to a yellow color, dominated by the halogen illumination

source. Similarly, in Figure 4(b), when φ=90° for the bilayer grating, the bottom layer is activated resulting in a spectral transmittance lineshape matching that of the monolayer grating with a higher color saturation due to increased refractive index variations in the bilayer structure. In contrast, at φ=0°, the top layer of the bilayer grating is activated, giving rise to a blue structural color which clearly differs from the φ=0° response observed for the monolayer grating.

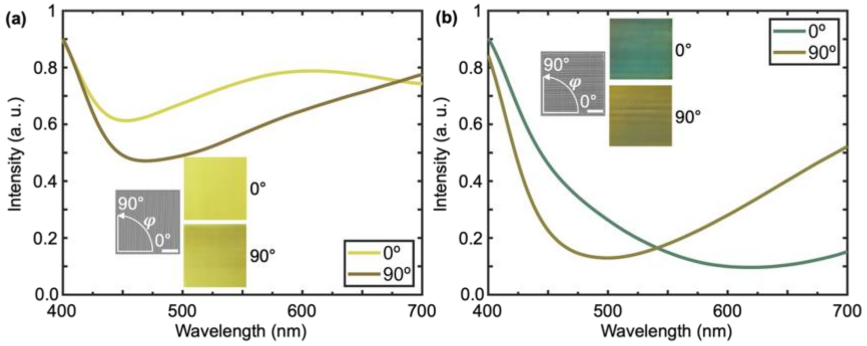

Figure S5. φ-dependent color responsiveness of grating structures. Transmittance spectra of (a) a monolayer (d=322 ± 15 nm and Λ=1.0 µm) and (b) a bi-grating (d=907 ± 21 nm and Λ=1.1 µm) at φ of 0° and 90° under LP incident light. Insets show SEM and optical microscope images of structural color responses. Scale bar: 10 µm.

References

[1] J. Schanda, "CIE Colorimetry," in *Colorimetry*, 2007, pp. 25–78.

[2] T Smith and J Guild, "The C.I.E. colorimetric standards and their use," *Trans. Opt. Soc.*, vol. 33, no. 3, p. 73, 1931, doi: 10.1088/1475-4878/33/3/301.

[3] International Comission on Illumination (CIE), "Colorimetry," 2004.

[4] Wolfram Research, "ImageMeasurements, Wolfram Language function," 2012. https://reference.wolfram.com/language/ref/ImageMeasurements.html.

[5] Wolfram Research, "RGBColor, Wolfram Language function," 1988. https://reference.wolfram.com/language/ref/RGBColor.html.

[6] Wolfram Research, "ChromaticityPlot, Wolfram Language function," 2014. https://reference.wolfram.com/language/ref/ChromaticityPlot.html.

[7] Wolfram Research, "WhitePoint, Wolfram Language function," 2014. https://reference.wolfram.com/language/ref/WhitePoint.html.
